# RP-HPLC Method for the Estimation of Dutasteride in Tablet Dosage Form

**DOI:** 10.4103/0250-474X.62247

**Published:** 2010

**Authors:** Dipti B. Patel, N. J. Patel, S. K. Patel, A. M. Prajapati, S. A. Patel

**Affiliations:** S. K. Patel College of Pharmaceutical Education & Research, Department of Pharmaceutical Chemistry, Ganpat University, Kherva-382 711, India

**Keywords:** Benign prostatic hyperplasia, dutasteride, RP-HPLC

## Abstract

A simple, sensitive and precise RP-HPLC method was developed for the determination of dutasteride in tablet dosage form. The RP-HPLC separation was achieved on phenomenex C_18_ column (250 mm, id 4.6 mm, 5 μm) using mobile phase methanol:water (90:10 v/v) at a flow rate of 1 ml/min at an ambient temperature. Quantification was achieved with photodiode array detection at 235 nm over the concentration range 1-12 μg/ml. The method was validated statistically and was applied successfully for the determination of dutasteride in tablets.

Dutasteride (DTS), selective inhibitor of both, type 1 and type 2 isoforms of 5α-reductase (5-AR) enzyme that converts testosterone to 5α-dihydrotestosterone (DHT) which is responsible for enlargement of prostate, is used in treatment of benign prostatic hyperplasia, frequently occurring in men over the age of 50 years[1]. Chemically, DTS is (5α,17β)-N-{2,5 bis (trifluoromethyl)phenyl}-3-oxo-4-azaandrost-1-ene-17-carboxamide with an empirical formula C_27_H_30_F_6_N_2_O_2_, representing a molecular weight of 528.5 g/mol[2]. Literature survey revealed LC-MS and HPLC methods for estimation of DTS in human plasma and pharmaceutical dosage forms[3–5]. A LC-MS-MS method is reported for the simultaneous determination of tamsulosin and dutasteride in human plasma[6]. So it was thought of interest to develop a simple and sensitive RP-HPLC method for determination of DTS in tablet.

All the reagents used were of HPLC grade and analytical grade. Reference standard of DTS was procured from Intaas Pharmaceutical Limited, Ahmedabad with 99.98% purity. Tablets of two different batches of Veltride (0.5 mg) of Intaas Pharmaceutical Ltd. were purchased from a local pharmacy. A standard stock solution of DTS (1 mg/ml) was prepared by dissolving 25 mg of drug in 25 ml methanol. Working standard solution (100 μg/ml) was prepared from stock solution by proper dilution with methanol.

A Shimadzu HPLC (LC-2010HT-liquid chromatograph) equipped with PDA detector (SPD-M20A), phenomenox (Torrance, CA) C_18_ (250×4.6 mm i.d., 5 μm) column and LC solution software were used. The mobile phase used was methanol:water (90:10, v/v) which was filtered through nylon 0.45 μm membrane filter and degassed by ultrasonication for 15 min.

Linearity of the method was investigated by serially diluting the working standard to give a concentration range of 1-12 μg/ml and 20 μl from this solution was injected. The flow rate was maintained at 1 ml/min. Temperature of the column was kept at ambient and the effluent was monitored at 235 nm. Calibration curve was constructed by plotting concentration against peak area. The method was validated for linearity, precision, accuracy, and specificity, limit of detection and limit of quantification as per ICH guidelines[7].

Assay of tablets of DTS were performed. Thirty tablets of each batch having 0.5 mg strength were weighed and ground to a fine powder. A quantity of tablet powder equivalent to 10 mg of DTS was transferred to 10 ml volumetric flask, dissolved and diluted with methanol to obtained 1 mg/ml. The solution was sonicated for 15 min and filtered through 0.45 μm membrane filter. The solution was further diluted to obtain concentration 10 μg/ml. Peak area of the above prepared tablet solutions of DTS were measured by using above mentioned chromatographic conditions and the amount of DTS were found from regression equation.

To optimize the HPLC parameters, several mobile phase compositions were tried. Various mobile phases having different ratios of methanol, water and acetonitrile were tried. Drug was retained in mobile phase consisting of acetonitrile:water (60:40, v/v) and methanol:water (60:40, v/v). In acetonitrile:water (90:10, v/v) tailing in the peak was observed. Good peak symmetry and satisfactory retention time was obtained with mobile phase consisting of methanol:water (90:10 v/v). Quantification was achieved with PDA detection at 235 nm based on peak area. The retention time of DTS obtained was 5.24±0.112 (fig. 1). The system suitability tests for HPLC were carried out on freshly prepared solution of DTS (10 μg/ml) and the parameters were studied. The results were summarized in Table 1.

**Fig. 1:**
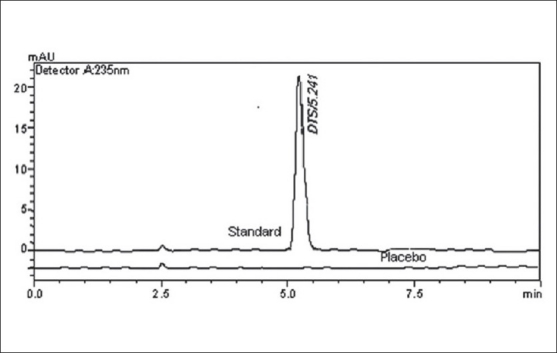
Overlay chromatogram of standard dutasteride (10 μg/ml) and placebo

**TABLE 1:** SYSTEM SUITABILITY TEST PARAMETERS

Parameters	RP-HPLC method
Retention time, min	5.241±0.112
Tailing factor	1.048±0.274
Asymmetry factor	1.121±0.510
Theoretical plates	5859±0.774
Resolution	2.895±0.431

^a^RSD is relative standard deviation.

The linear regression data showed a good linear relationship over the concentration range of 1-12 μg/ml as summarised in Table 2. The limit of detection (LOD) and the limit of quantification (LOQ) of the drug were found by scanning the solution of DTS having different lower concentrations and the LOD and LOQ were found to be 0.5 and 1 μg/ml indicates that the method is sensitive (Table 2). The intraday and interday precision were determined by analyzing standard solution of DTS at three different concentration levels (6, 8, 10 μg/ml). The % RSD for intraday and interday precision was found to be 0.257–0.712% and 0.438-1.080% respectively which indicate that the method is precise (Table 2). Repeatability of the method was studied by injecting 10 μg/ml solution of DTS for six times and peak area was measured and % RSD was calculated which was found to be 0.195 shows repeatability of the method (Table 2). Accuracy of the method was evaluated by standard addition method in which appropriate portions of stock solutions of DTS were spiked into blank placebo matrix to produce concentrations of 80, 100 and 120% of the theoretical concentration. The mean recovery of spiked samples obtained was in range of 98.87-100.31% reveals no interference of excipients and shows that the method is accurate. The proposed validated method was successfully applied to determine DTS in tablet form. The results obtained for tablets of DTS were comparable with the corresponding labelled amounts (0.5 mg/tab) (Table 3). Robustness of the method was estimated by changing the mobile phase composition (3±3), wave length±1 nm, injection volume (20±2 μl), column temperature (40±3°) and RSD values for all these changes calculated were less than 2 indicate that proposed method is robust. The proposed RP-HPLC method was accurate, precise, sensitive and rapid. The method also can be extended for the routine analysis of DTS in tablet dosage form.

**TABLE 2:** REGRESSION CHARACTERISTICS AND VALIDATION PARAMETERS

Parameters	Dutasteride
Linearity range (μg/ml)	1-12
Correlation coefficient (r^2^)	0.9985
Regression equation (y= mx+c)	
Slope (m)	131078
Intercept (C)	−25526
LOD^a^ (μg/ml)	0.5
LOQ^b^ (μg/ml)	1
Accuracy (%)	98.87-100.31%
Repeatability (RSD^c^, %, *n* = 6)	0.195
Precision (RSD, %)	
Interday (*n* = 3)	0.438-1.080%
Intraday (*n* = 3)	0.257-0.712%

a LOD is limit of detection

b LOQ limit of quantification and

c RSD is relative standard deviation

**TABLE 3:** ANALYSIS OF TABLETS OF DUTASTERIDE

Formulation	Label claim (mg/tab)	% of label Claim*±SD	% CV
Tablet 1	0.5	99.48 ± 1.367	0.793
Tablet 2	0.5	99.64 ± 0.869	1.040

* Mean of three determinations, SD is standard deviation and CV is coefficient of variation
